# Controlling M-A Constituents and Bainite Morphology for Enhanced Toughness in Isothermally Transformed Low-Carbon Ni-Cr-Mo Steel

**DOI:** 10.3390/ma18091945

**Published:** 2025-04-24

**Authors:** Guang Ji, Dianfu Fu, Guangyuan Wang, Kaihao Guo, Xiaobing Luo, Feng Chai, Tao Pan

**Affiliations:** 1 School of Mechanical Engineering, Nantong Institute of Technology, Nantong 226002, China; 2 Division of Structural Steels, Central Iron and Steel Research Institute, Beijing 100081, China; 3 Engineering Research & Design Institute, CNOOC Research Institute Co., Ltd., Beijing 100029, China

**Keywords:** isothermal transformation, bainite, transformation kinetic, low-temperature toughness, M-A constituents

## Abstract

The isothermal bainitic transformation kinetics, microstructure, and mechanical properties of the quenched low-carbon high-strength steel have been investigated via dilatometric measurements, microstructural characterization, and mechanical tests. The results show that the pre-transformed isothermal bainite promotes martensitic transformation, increasing the martensitic transformation temperature, and enhancing the transformation rate. The microstructure of the 400 °C isothermal steel consists predominantly of lath bainite ferrite with dot/slender M-A constituents, whereas the steel treated at 450 °C contains a combination of martensite/lath bainite and granular bainite. The presence of massive M-A constituents contributes to brittle fracture as these constituents tend to promote crack initiation. Hence, the 450 °C treatment, which leads to the formation of massive M-A constituents, induces brittleness, while the finer M-A constituents formed at 400 °C exert minimal influence on the toughness and result in a more stable microstructure owing to their small size and the surrounding fine lath microstructure. The differences in microstructure and properties between the steels treated at 400 °C and 450 °C illustrate the importance of controlling the quenching cooling rate in the high-temperature bainitic transformation region during thick plate quenching processes.

## 1. Introduction

Low-carbon high-strength heavy steels are widely used in marine platforms, gas storage tanks, and other structural applications. These heavy plates are typically required for their high strength, excellent low-temperature toughness, and good weldability [1]. Achieving good hardenability is crucial for ensuring the toughness and strength of heavy plates, which depends on the optimal combination of quenching strategy and alloying element addition [2]. However, due to the effects of heat transfer from the interior and latent heat release during phase transformation, local isothermal or even reheating behavior occurs invariably near the center of the plates [3]. This stage is similar to the isothermal treatment of the local area of the plates (typically close to the center of the plate) and usually lasts for more than one minute around the bainitic transformation point [3,4]. Consequently, a complex microstructure consisting of martensite (M), granular bainite (GB), and lath bainite (LB) is generated, which has a destructive influence on the microstructural homogeneity and hardenability of the steel [5]. Therefore, studying the isothermal bainitic transformation in low-carbon high-strength steel is essential for understanding the evolution of microstructure and properties across the cross-section of heavy plates and for optimizing the quenching strategy for low-carbon high-strength heavy plates.

Bainite transformation is a diffusion and coherent transformation resulting from undercooled austenite transformation [6,7]. The supersaturation of the bainite sub-unit is relieved either in the form of carbide precipitation or by the diffusion of carbon to austenite. The diffusion of carbon in austenite plays an important role in determining the rate of growth of the bainitic laths and the morphology of bainite. Building on this knowledge, previous research has focused on designing bainitic structures to enhance material properties by controlling isothermal temperature and holding time similar to the quenching and partitioning (QP) treatment for steels [8,9]. Liu investigated the isothermal bainite transformation of LSFed 300M steel at different temperatures, demonstrating that the introduction of lower bainite formed at lower transformation temperatures is beneficial for enhancing toughness without significantly sacrificing strength [8]. An increase in the lower bainite content results in a finer multiphase microstructure due to the dividing effect on the prior austenite grains by bainite ferrite, and the stable film-like retained austenite [9,10]. In addition, the strategy of quenching to the martensite transformation range and partitioning in the bainite transformation range to obtain pre-transformed martensite and ultrafine lower bainite has been proposed to optimize the mechanical properties of QP steels [11,12].

Attentively, M-A constituents and granular bainite transformed at high temperatures within the bainite transformation range have been widely recognized as detrimental microstructures and reduce the toughness of steels [13]. M-A constituents are essentially a mixture of untempered martensite embedded in carbon-enriched retained austenite that is formed due to incomplete bainite transformation [14,15]. Either the cracking of M-A constituents or the debonding of coarse M-A constituents from the matrix can be effective initiation sites for catastrophic brittle fracture, especially for necklacing M-A constituents along coarse prior austenite grain (PAG) boundaries [16,17]. Previous studies have also reported that small M-A constituents are beneficial to the improvement of impact toughness [18,19,20]. The structure and morphology of M-A constituents are determined by pre-transformed bainite ferrite and the kinetic characteristics of carbon diffusion [7,15]. Hence, an improved bainitic transition rate and limited geometric space, achieved by decreasing the transition temperature, accelerating the cooling rate, and reducing the PAG size, restrict the coarsening of M-A constituents [17,21,22,23]. Therefore, in order to obtain superior toughness, an appropriate quenching process should prevent stasis in a specific range of temperatures to generate coarse M-A components. However, the influence of isothermal transformation on the microstructure and properties of Ni-Cr-Mo heavy steel has not been systematically studied, and it is unclear how the microstructure transforms during cooling and isothermal processes and how these components, including M-A constituents, affect the low-temperature toughness of steels.

In this study, the kinetics of isothermal bainite transformation and the impact of pre-transformed bainite on the kinetics of martensitic transformation were probed by designing two isothermally transformed steels that have different isothermal temperatures within the bainite transformation range and comparing the results with the non-isothermal steels. Furthermore, the microstructural characteristics of the isothermally transformed bainite and its effect on low-temperature toughness have been systematically investigated using multi-characterization techniques, with a particular focus on the size and structure of M-A islands in different isothermal steels.

## 2. Materials and Methods

The Fe-0.08C-0.3Si-0.8Mn-5(Ni + Cr + Mo)-0.1V-0.03Al (wt. %) steel was produced by casting, electroslag remelting, and hot rolling process. In order to investigate the influence of isothermal treatment on microstructure and low-temperature toughness, the Gleeble-3800 thermomechanical simulator from DSI (Dynamic System Inc., Poestenkill, NY, USA) was used. A rectangular specimen with dimensions of 10.5 × 10.5 × 65 mm was extracted from the central region of a 20 mm thick hot-rolled plate for the purpose of heat treatment and testing. The bainitic transformation temperature range of the experimental material is between 500 °C and 400 °C. Therefore, the as-rolled steel was first heated to 880 °C at a rate of 10 °C/s and kept for 10 min, then cooled to 450 °C (named ISO-450 steel) and 400 °C (named ISO-400 steel) at a rate of 10 °C/s and held for 120 s, respectively. After the isothermal treatment, the specimens were cooled to room temperature at a rate of 2 °C/s. Then, to compare the toughness of isothermally and non-isothermally treated steels, two specimens were cooled to room temperature from 880 °C at a rate of 10 °C/s (named CC-10 steel) and 2 °C/s (named CC-2 steel), respectively. In addition, the transformation kinetic analysis was based on the dilatometry curves obtained by the compression module of the dilatometer (DIL 805A/D, TA Instruments, New Castle, DE, USA). In order to mitigate the influence of the rolling process on the dilatometry curves, cylindrical specimens with a diameter of 4 mm and a length of 12 mm were machined from rectangular samples that had been quenched at 900 °C.

The microstructure information was characterized by a scanning electron microscope (SEM, model: Quanta650 FEG, FEI Company, Hillsboro, OR, USA) equipped with an Oxford electron backscattered diffraction (EBSD) detector. The microstructural morphology was characterized at an accelerating voltage of 5 kV. EBSD analysis was carried out at an accelerating voltage of 20 kV, with a scan area of 200 × 200 μm^2^ and a step size of 0.2 μm. Aztec crystal 2.1 software was employed to analyze the crystallographic feature of the bainite. The thin foils were electro-polished at −20 °C in a 6% perchloric acid solution, and then further examined under a JSM-7900F field emission transmission electron microscope, JEOL Ltd., Tokyo, Japan. The microstructural development during the 450 °C isothermal stage was tracked in situ by high-temperature laser-scanning confocal microscopy (LSCM). The volume fraction of retained austenite was measured using a D8 ADVANCE X-ray diffractometer, Bruker Corporation, Billerica, MA, USA (Cu Kα radiation, λ = 1.5418 Å) at a scan rate of 2°/min, over a 2θ range of 30° to 90°.

**Figure 1:**
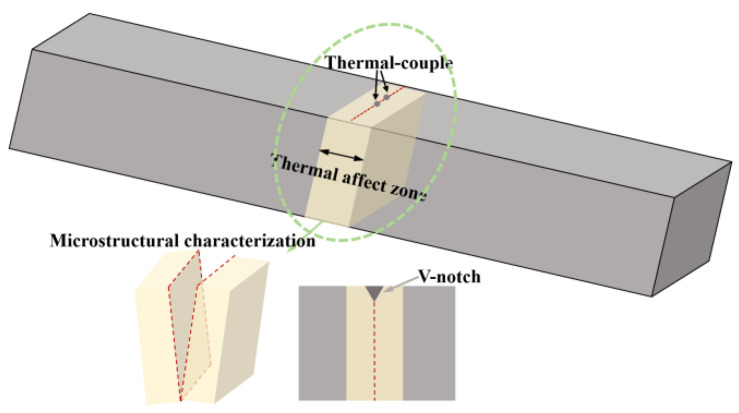
Schematic Diagram of Specimen Processing.

The impact tests were performed with standard Charpy V-notch specimens at the temperatures of −80 °C and −60 °C. The Vickers hardness testing was measured at five different locations on each specimen using a Buehler Micro-hardness tester (Illinois Tool Works (ITW), Lake Bluff, IL, USA) under an applied load of 98 N, and the reported value represents the average of these five indentations. The sections chosen for microstructural characterization and impact notch machining are shown in Figure 1.

## 3. Results

### 3.1. Transformation Kinetic

**Figure 2:**
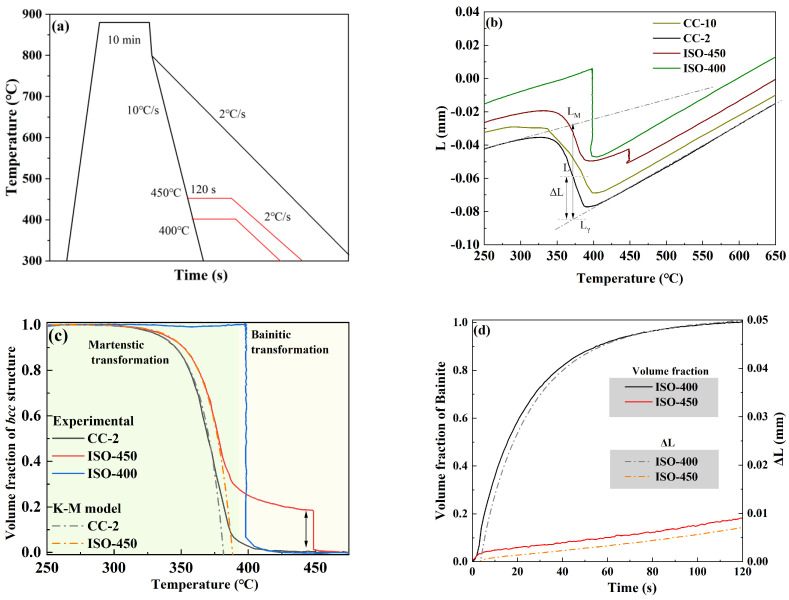
Kinetic analysis for the martensite transformation: (**a**) the diagrammatic sketch describes the isothermal and non-isothermal cooling curves; (**b**) the dilatometry curves during cooling; (**c**) the bcc structure volume fraction vs. temperature; (**d**) the bcc structure volume fraction vs. holding time.

Figure 2a presents the diagrammatic sketch of the thermal cycle, and Figure 2b exhibits the dilatometry response obtained during cooling for these specimens. Based on the analysis of the dilatometry curves of the CC-10 steel, the experimental start temperature of martensitic transformation (*M*_S_) was defined as 397 °C, at which a martensite volume fraction of 0.02 was formed. For the ISO-400 steel, the bainite transformation is practically complete during the isothermal holding at 400 °C, and there is no apparent volume expansion below *M*_S_. The PAGs in the ISO-450 steel gradually transformed to bainite during isothermal holding at 450 °C, then slowly transformed to bainite during further cooling, and rapidly transformed to martensite when the temperature dropped below the *M*_S_. The volume fraction of bainite or martensite was obtained by applying the lever rule to the average dilatometry curves and assuming that only martensitic transformation occurred below the *M*_S_. The microstructure of the ISO-400 specimen is fully composed of isothermal bainite, whereas that of the ISO-45 specimen consists of approximately 20% isothermal bainite and 80% martensite. The Koistinen–Marburger (KM) model can be used to study the influence of the isothermal bainitic transformation on the kinetics of martensite formation:

(1) $$f_{\alpha^{\prime }}=1-\exp[-\alpha_{m}(T_{KM}-T)]$$

where *f*_α_ is the martensite volume fraction, *T_KM_* is the Koistinen–Marburger martensite start temperature, and α*_m_* is the overall rate parameter. Equation (1) was fitted to the experimental curves. Since the KM model is not applicable for the early stages of the transformation and the bainitic transformation occurred before the martensitic transformation of the ISO-450 steel, data above a martensite fraction of 0.4 were adopted in the fitting. Here, the overall rate parameters of the CC-2 steel and ISO-450 steel are 0.0630 K^−1^ and 0.0650 K^−1^, respectively, indicating that the martensite transformation rate increased due to the pre-transformed bainite. The martensitic transformation rate is affected by factors such as transformation temperature and composition. As carbon is ejected to the untransformed austenite during the bainite transformation, the *M*_S_ of the untransformed austenite should be improved. Nevertheless, previous studies have shown that stress can accelerate both bainite and martensite transformation rates, suggesting that pre-formed bainite causes austenite deformation and promotes martensitic transformation [24,25]. It has been reported that the pre-transformed martensite will provide more nucleation sites for bainite [11]. However, whether pre-transformed bainite can provide nucleation sites for bainite is still unknown. Figure 2d shows how the bainite volume fraction climbed as the holding time increased. When held at 400 °C, bainite underwent an explosive transformation, and at 40 s, 80 percent of it had formed. Compared to -the ISO-400 steel, the bainite transformation at 450 °C proceeds slowly through the isothermal process, growing almost linearly up to 20%. The decrease in bainite transformation temperature will increase the activation energy for bainite transformation, thereby increasing the bainite transformation rate, which is consistent with previous research [26].

### 3.2. Microstructural Feature

**Figure 3:**
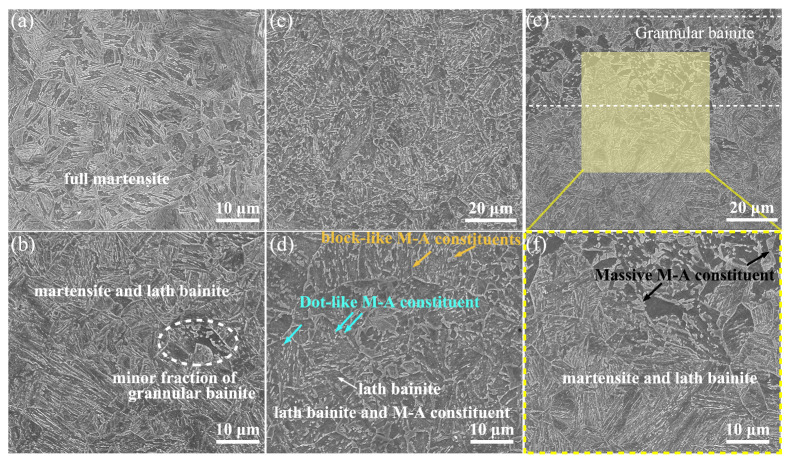
SEM maps for the isothermal and non-isothermal steels: (**a**) CC-10 steel; (**b**) CC-2 steel; (**c**,**d**) ISO-400 steel; (**e**,**f**) ISO-450 steel.

Figure 3 shows the SEM maps of the isothermal and non-isothermal transformed steels. CC-10 consists entirely of martensite, while CC-2 consists of martensite and a small fraction of bainite. The transformation point of this bainite was not found in the dilatometry curve of CC-2 steel, which is probably acceptable as the dilatometer sensitivity is about 10 percent volume fraction transformed. Compared to full martensite, the mixture of martensite and lath bainite (M/LB) normally exhibits better toughness along with excellent strength and is extensively applied in offshore steels. Further, large amounts of island-like structures were observed in the isothermally transformed steels, which were deemed to be the M-A constituents transformed from the C-rich residual austenite. The ISO-400 steel is entirely composed of lath bainite ferrite as the lath boundaries were decorated by dot/slender M-A constituents (indicated with arrows in Figure 3d). In contrast to the ISO-400 steel, the ISO-450 steel is composed of two different morphologies—one is the mixture of lath bainite and martensite, and the other is the granular bainite. According to the statistical results derived from the SEM images, the volume fraction of GB in the ISO-400 steel is estimated to be around 10%. In addition, the M-A constituents in the ISO-450 steel exhibit a larger size than those in the ISO-400 steel.

**Figure 4:**
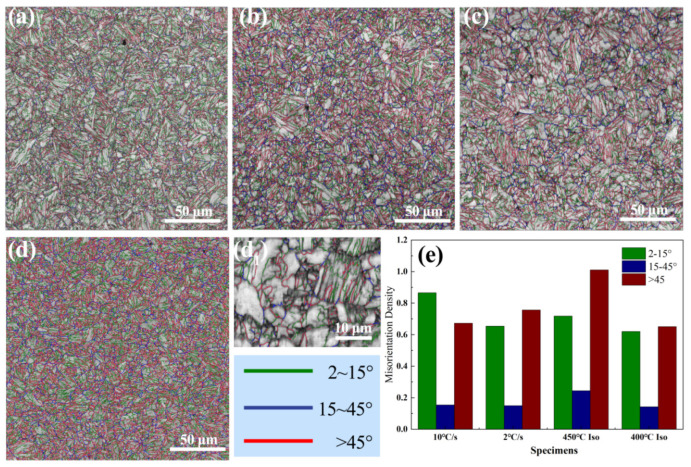
The band contrast (BC) map overlaps the grain boundary map depicting boundary distribution for (**a**) CC-10 steel, (**b**) CC-2 steel, (**c**) ISO-400 steel, and (**d**,**d_1_**) ISO-450 steel. (**e**) The boundary density of different misorientations for the steels.

Figure 4 shows the band contrast morphology and the grain boundary (GB) distribution of the steels. The non-isothermal steels and the ISO-400 steel exhibit a lath-like morphology, which is in accordance with the SEM maps. The ISO-450 steel also exhibits a lath-like morphology, while massive bainite ferrite was observed in the local region of the ISO-450 steel, as shown in the enlarged map in Figure 4d_1_. In addition, island-like M-A constituents consist of unindexed pixels or pixels indexed as BCC, with the lower BC being observed in Figure 4d_1_. The M-A constituents normally consist of substructures like martensite variants and austenite grains. These martensite variants transformed from the C-rich austenite and maintained the K-S orientation relationship with their neighboring retained austenite [27]. The grain boundary (GB) misorientation, as obtained from the EBSD analysis, is presented in Figure 4e. The density of high-angle boundaries (HAGBs, >45°) was at its maximum in the ISO-450, followed by the CC-2, CC-10, and ISO-400. The density of low-angle boundaries (LAGBs, <15°) was at its maximum in CC-10. The higher density of the HAGBs in the CC-2 steel can be attributed to the refinement of microstructure caused by the geometrical partitioning of the prior austenite grain by pre-transformed bainitic ferrite. The strong variant selection (the V1&V4 variant pair) and large amounts of defects like dislocations induced by fast quenching rate generate a higher density of LAGBs in CC-10 steel. Granular bainite in the ISO-450 steel is typically characterized by coarse bainitic grains, however, it exhibits the maximum density of HAGBs. This phenomenon is explicable as the fraction of granular bainite only accounts for 10% and numerous M-A constituents also contribute to the increase in the density of HAGBs.

**Figure 5:**
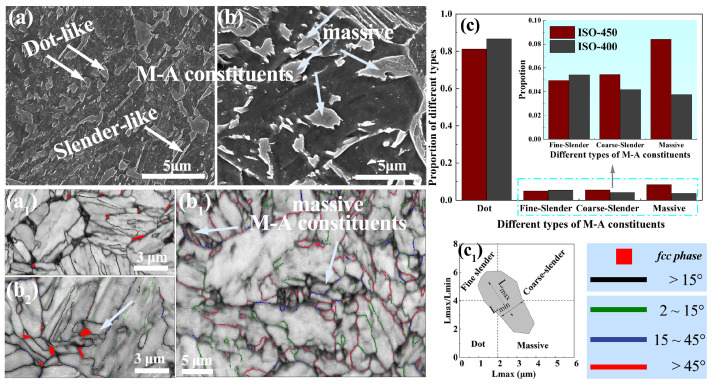
The characterization of the morphology and structure of the M-A constituents: (**a**,**b**) SEM images showing the morphology of M-A constituents in the ISO-400 steel (**a**) and the ISO-450 steel (**b**), respectively; (**a_1_**) EBSD results for M-A constituents in the ISO-400 steel, revealing the substructure within the M-A constituents; (**b_1_**,**b_2_**) EBSD results for M-A constituents in the ISO-450 steel, revealing the substructure within the M-A constituents; (**c**) statistical information on the distribution of different types of M-A constituents; (**c_1_**) the size limit used to classify the different types of M-A constituents.

A systematic characterization of the M-A constituents is presented in Figure 5. The size and structure of the M-A constituents transformed in the isothermal stage were dominated by the transformation temperature—Figure 5a,b exhibits the morphology of the M-A constituents in the ISO-400 steel and ISO-450 steel, respectively. There are large amounts of massive M-A constituents in the ISO-450 steel, both the maximum size (L_max_) and the minimum size (L_min_) of these M-A constituents exceed 2 μm. Compared to those in the ISO-450 steel, the M-A constituents in the ISO-400 steel mainly exhibit the dot and fine–slender morphologies. Statistical information on the number and density of the various M-A constituent types in the ISO-450 and ISO-400 steel is displayed in Figure 5c. It demonstrates that the number and density of massive M-A components in the ISO-450 steel is double that of the ISO-400 steel. Figure 5b_1_ is a BC map that overlaps the grain boundary map, showing that the massive M-A constituents consist of various martensite variants and that these martensite sub-grains are separated by high-angle grain boundaries. Figure 5a_1_,b_2_ are overlaid with the fcc-phase map. Figure 5b_2_ shows that the massive M-A constituents in the ISO-450 steel contain block-like austenite grains, with a significantly larger size and higher fraction compared to the austenite grains in the M-A constituents of the ISO-400 steel. Some austenite grains in the ISO-450 steel reach sizes approaching 1 μm. In contrast, the M-A constituents in the ISO-400 steel exhibit a significant reduction in size, along with noticeably decreased martensite size and increased lattice distortion. The austenite content in the M-A constituents of the ISO-400 steel is very low, and austenite films were observed at the grain boundaries.

**Figure 6:**
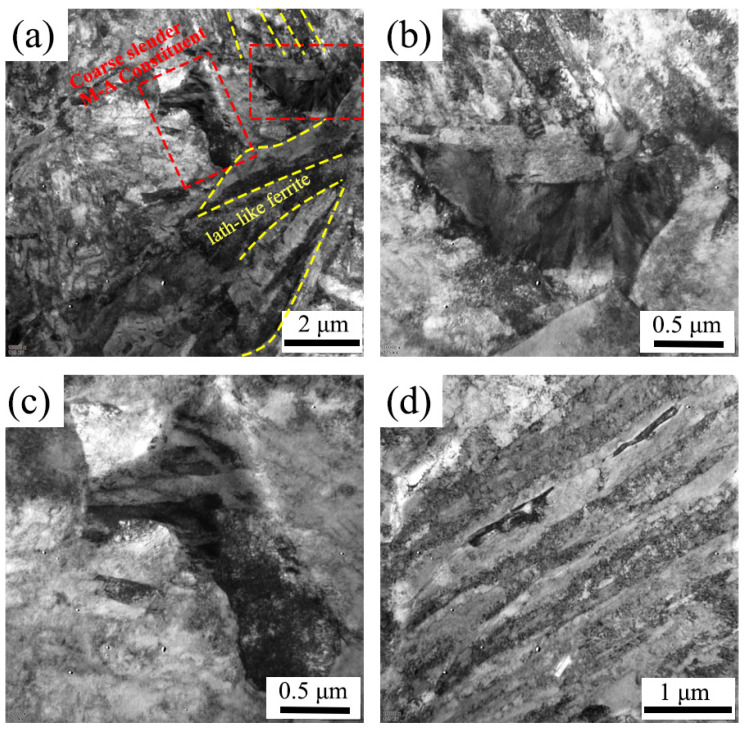
The TEM results of the ISO-400 steel: (**a**) the morphology of M-A constituents; (**b**,**c**) the enlarged maps of the local area in (**a**); (**d**) the morphology of lath-like isothermal bainite ferrite.

**Figure 7:**
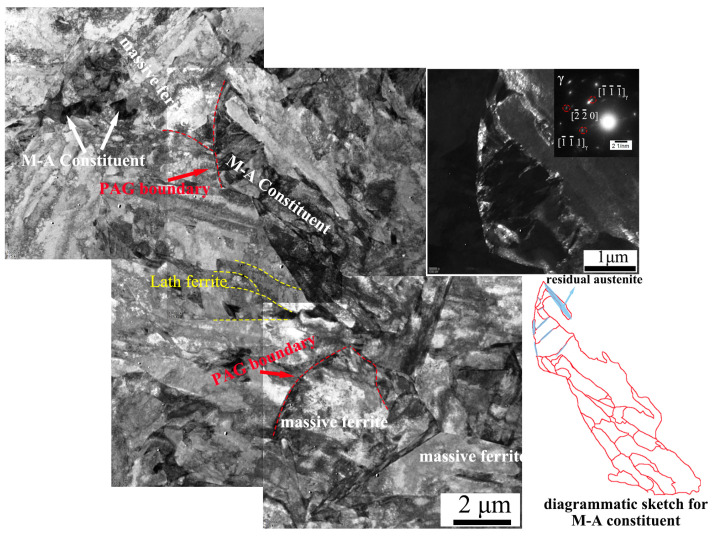
A TEM map for the M-A constituents in the ISO-450 steel.

Figure 6 and Figure 7 present the TEM images of M-A constituents in the isothermal transformed steels treated at 450 °C and 400 °C, respectively. Figure 6d exhibits the bainitic morphology of the ISO-400 steel, confirming that the ISO-400 steel is primarily composed of lath-shaped bainitic ferrite. The M-A constituents in the ISO-400 steel are smaller in size compared to those in the ISO-450 steel. These constituents contain martensite sub-grains smaller than 100 nm and exhibit a high density of dislocations, which enhances the hardness of the M-A constituents in the ISO-400 steel. Additionally, fine bainitic laths are distributed around the M-A constituents in the ISO-400 steel. In contrast, the massive M-A constituents at the prior austenite grain boundaries in the ISO-450 steel consist of a random arrangement of martensite and austenite, which is also named the type-III M-A constituent according to its structure [28]. The dark-field images reveal that the austenite is located at the edges of M-A constituents and martensite lath boundaries. And, the M-A constituents in the ISO-450 steel are surrounded by blocky bainitic ferrite and several lath-shaped bainitic ferrites, consistent with the EBSD results.

**Figure 8:**
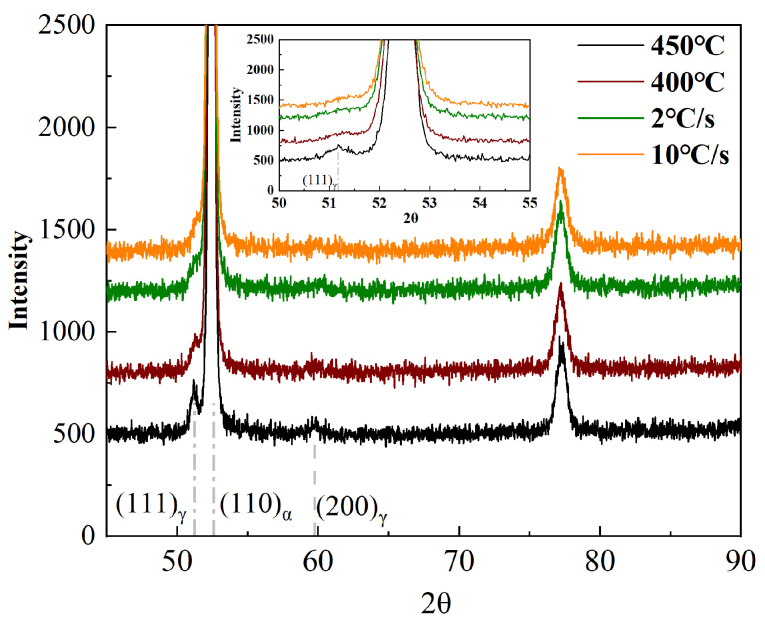
The XRD pattern for the different steels.

To further clarify the austenite fraction in the steels, XRD patterns were utilized for analysis, as shown in Figure 8. Although the austenite fraction below 2% cannot be accurately measured by XRD, a significantly increased intensity of the {111}_γ_ peak indicates a significant increase in the austenite fraction. In fact, considering that the ISO-450 steel contains only 20% bainite, the austenite fraction within the M-A constituents of the ISO-450 steel is much higher than that of the ISO-400 steel. During the bainitic transformation, the carbon will be excluded into the surrounding austenite grains from the bainitic ferrite. A higher isothermal temperature enhances the carbon diffusion rate, further stabilizing the retained austenite. Additionally, based on the Williams equation, the dislocation densities of CC-10 steel, CC-2 steel, ISO-450 steel, and ISO-400 steel were calculated as 6.1253 × 1011, 5.9233 × 1011, 4.1972 × 1011, and 4.9567 × 1011 cm^−2^, respectively.

**Figure 9:**
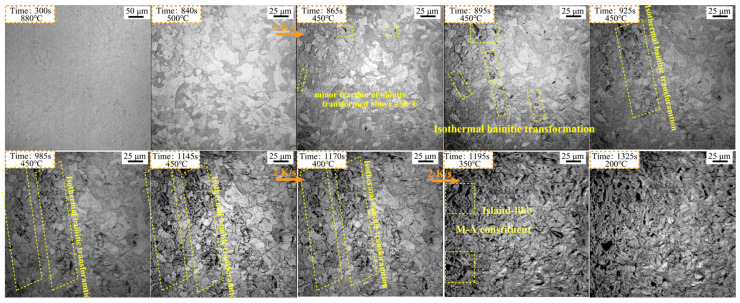
In situ observations of transformed microstructures in ISO-450 steel.

Using the high-temperature LSCM observation, the real-time features of bainite at the early stage of the transformation were recorded, as shown in Figure 9. The phase transformation does not occur when the temperature is above 500 °C. The minor fraction of bainitic ferrite transformed above 450 °C as signified in Figure 9. However, this temperature is higher than that detected using a dilatometer (Figure 2a). This discrepancy is caused by the sensitivity of the dilatometer, and is similar to the discovery of a small fraction of bainite in CC-2 steel. With a further holding time at 450 °C, bainite gradually transformed. Nevertheless, the bainite formation is inhomogeneous at the grain scale since the remaining grains are still isolated from the transformation region. The remaining austenite grains changed instantly, indicating the start of martensitic transformation, when the temperature dropped below 400 °C. This result is in good agreement with the SEM findings shown in Figure 3e. The pre-transformed granular bainite was sandwiched between the lath structures. Meanwhile, it is very interesting to find that a newly formed island-like M-A constituent was observed among the pre-transformed bainitic ferrite, as marked with a cycled yellow square.

According to previous studies, the formation of bainitic ferrite is thought to be displacive, and the carbon partitioning into the remaining austenite happens right after each bainite platelet grows. The bainite transformation at a high temperature is slow, and no apparent explosive bainite transformation occurs. As undercooling increases and carbon diffusion proceeds, the compositional heterogeneity of austenite grains increases, forming C-rich and C-poor regions, which in turn promotes bainite transformation. The increased cooling rate shortens the holding time at high temperatures, suppressing bainite transformation and restricting it within a small amount of unstable austenite. When held at 450 °C, bainitic ferrite preferentially forms in regions with lower concentrations of alloying elements. The carbon partitioning during the isothermal process is the primary cause of the formation of island-like structures in the bainitic microstructure. The bainitic ferrite formed at 450 °C expels carbon into the surrounding austenite as it grows. These C-enriched austenite grains wrapped by pre-transformed bainite ferrite partially transform into C-enriched martensite and residual austenite (ISO-450 specimen), or completely transform into C-enriched martensite (ISO-400 specimen) according to the different *M*_S_ points of the residual austenite.

The difference in the morphology of bainitic ferrite results from the variant selection of bainite. Previous research has indicated that low-temperature bainite transformation requires a greater number of V1&V2 variant pairs to accommodate the strain induced during the transformation process [29]. At a high transformation temperature, however, bainite transformation primarily relies on the self-accommodation of austenite grains, leading to the elevated fraction of V1&V4 or V1&V1 variant pairs [30]. The V1&V2 variants are associated with high-angle grain boundaries, approximately 60°, whereas the V1&V4 variants correspond to small-angle grain boundaries, around 10°. The variant selection is responsible for the differences in the misorientation distribution.

The morphology of M-A constituents is strongly affected by the shape and crystallography of the surrounding bainite. N. Takayama points out that M-A constituents are elongated along the growth direction or habit plane of the surrounding bainitic ferrite when the neighboring variants of bainitic ferrite share them [15]. Therefore, the transformation temperature of M-A constituents dominates the morphology of M-A constituents, although these M-A constituents were finally formed in the cooling to room temperature from the remaining austenite [31]. M-A constituents are basically formed at the lath boundaries or the prior austenite grain boundaries. The explosive bainitic transformation and refined bainitic laths, which limit the geometric space for M-A constituents, restrict the growth of massive M-A constituents and increase the fraction of dot-like M-A constituents, as the isothermal temperature decreases.

### 3.3. Low-Temperature Toughness

Table 1 presents the hardness and Charpy impact properties at temperatures of −60 °C and −80 °C. The hardness was at its maximum in the CC-10 steel (376 HV_10_), followed by CC-2, ISO-450, and ISO-400 steel. Compared to non-isothermally transformed steels, isothermally transformed steels exhibit a lower hardness. This is attributed to the relief of lattice distortion and the reduction in internal stresses during the isothermal process.

For low-temperature toughness, untempered martensite steels show significant internal distortion and a high density of defects, which always exhibit poor toughness. Compared to the full martensite steels (CC-10 steel), the martensite + lath bainite structure (CC-2) has a higher density of HAGBs, a significantly refined effective grain size, a reduced dislocation density, and an improved low-temperature toughness [32]. Regarding isothermally transformed steels, the low-temperature toughness of the ISO-450 steel is significantly reduced. Although the ISO-450 steel predominantly consists of lath-like structures with the highest density of HAGBs, these boundaries are partially contributed by massive M-A constituents, which do not effectively hinder crack propagation. Moreover, the massive M-A constituents will cause stress concentration, promoting crack initiation and growth. In contrast, the ISO-400 steel primarily comprises a ferritic matrix with lath morphology and relatively small M-A constituents. Small-sized M-A constituents have little impact on low-temperature toughness.

**Figure 10:**
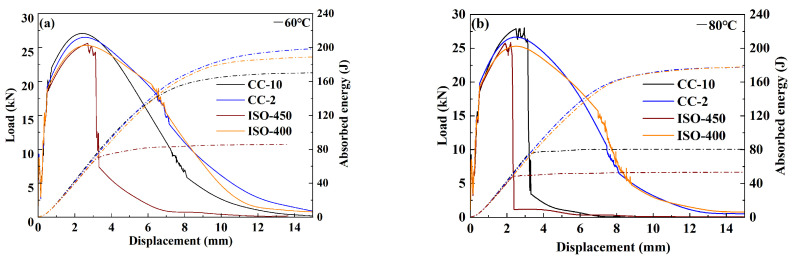
Load and absorbed energy versus displacement curves obtained from instrumented Charpy impact testing of experimental steels at (**a**) −60 °C and (**b**) −80 °C.

Figure 10 exhibits the load-deflection curves of the experimental steels at temperatures of −60 °C and −80 °C. Supersaturated as-quenched martensitic steels typically exhibit a high ductile–brittle transition temperature (DBTT) due to lattice distortion and internal stresses, as they lack a tempering process. However, the CC-10 steel shows better toughness compared to the ISO-450 steel. Under a −60 °C impact, the load of the ISO-450 steel deeply dropped after the peak load, demonstrating that once a crack initiates, it immediately adopts an unstable propagation mode until the crack tip blunting. The other steels, in contrast to the ISO-450 steel, proceed with a stable crack propagation mode after the peak load. Under a −80 °C impact, compared to other fractured steels, the crack in the ISO-450 formed earlier, and once the crack forms, it adopts a completely unstable propagation mode. The ISO-400 steel consistently maintains a stable crack propagation mode until the extending cracks become excessively sharp, leading to instability. However, as the crack tip blunts, it reverts to a stable propagation mode. The CC-2 sample exhibits the best toughness, with the smallest unstable propagation region, indicating that the resistance to crack propagation is enhanced.

**Figure 11:**
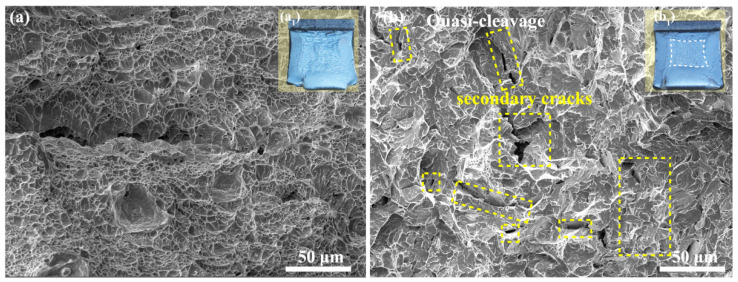
Fractographic analysis of Charpy impact specimens tested at −60 °C: (**a**) ISO-400 steel, with (**a_1_**) representing its macroscopic fracture morphology; (**b**) ISO-450 steel, with (**b_1_**) representing its macroscopic fracture morphology.

Figure 11 displays SEM fractographs of fractured Charpy specimens impact tested at −60 °C. The ISO-400 steel exhibits a completely ductile ‘fibrous-fracture appearance’, as characterized by the presence of multiple dimples on the fracture surfaces. In the ISO-450 steel, quasi-cleavage fracture accounted for approximately 45% of the fracture surface in addition to fibrous fracture. It is noteworthy that many secondary cracks were connected to the primary crack and extended laterally in the ISO-450 steel. Those secondary cracks possibly initiated around the brittle component located at grain boundaries, carbides, or M-A constituents. They nucleated in great quantities during loading, propagated rapidly, and either merged into the main crack or facilitated its propagation.

**Figure 12:**
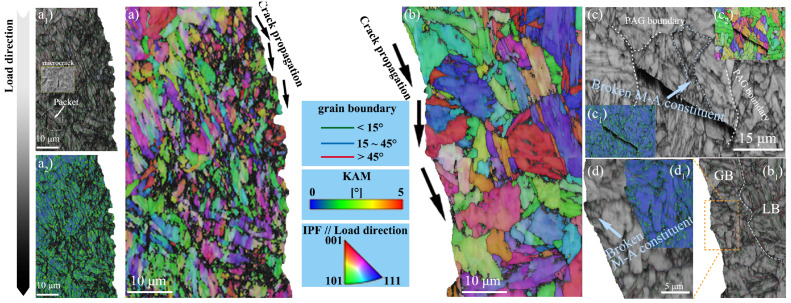
The fracture surfaces on the transverse cross-sections of the broken Charpy specimens characterized by EBSD: (**a**) the IPF map depicts the crack propagation of the ISO-400 steel; (**a_1_**) the BC map and (**a_2_**) KAM map of (**a**); (**b**) the IPF map depicts the crack propagation of the ISO-450 steel; (**b_1_**) the BC map of (**b**); (**c**) the BC map depicts the crack nucleation of the ISO-450 steel; (**c_1_**) the KAM map and (**c_2_**) the IPF map of (**c**); (**d**) the local enlarged BC map and (**d_1_**) KAM map of (**b_1_**).

To clarify the nucleation sites of the cracks, the EBSD micrographs showing the locations just beneath the fracture surfaces on the transverse cross-sections of the broken Charpy specimens are presented in Figure 12. The ISO-450 steel shows a straighter crack propagation path in the quasi-cleavage fracture region, as depicted in Figure 12b. The microstructure of the ISO-450 steel consists of M/LB and GB. This heterogeneity, resulting from the distribution of M/LB and GB, introduces microstructural plastic incompatibilities. GB exhibits lower strength and hardness compared to the M/LB. During impact loading, plastic deformation is predominantly localized in the GB, leading to stress concentration at the interface between GB and M/LB. Due to the heavy deformation of the bainitic ferrite matrix, high stress concentrates on the boundary of M-A and makes it crack or debond [16,33]. Crack initiation in the ISO-450 steel originates from broken M-A constituents, especially their massive morphology, as shown in Figure 12c. According to the calculation of the size of the Griffith crack based on Griffith theory, the width of the MA constituents can represent the initiation size of the cleavage crack [34]. Table 2 summarizes the numerical data of M-A constituents in the ISO-450 steel and ISO-400 steel. Although the ISO-400 sample exhibits a higher number density of M-A constituents, the majority are of the dot-type morphology, as illustrated in Figure 5c. In contrast, the number fraction of massive-type M-A constituents in the ISO-450 steel is approximately twice that observed in the ISO-400 steel. The average size of the M-A constituents in the ISO-450 steel exceeds 1 μm, and this value is widely recognized as a critical threshold associated with reduced fracture toughness. The increased number of coarse M-A constituents intensifies local stress concentrations around brittle phases and decreases the interspacing between microcracks, thereby contributing to crack initiation and propagation. In addition, the blocky austenite grains in the M-A austenite of the undeformed specimens were not observed near the fracture surface, indicating that the martensite transformation occurred in this residual austenite. The presence of deformation-induced martensite in the M-A constituents increases the brittleness tendency of the M-A constituents by intensifying the internal stress concentration within the M-A constituents or at their boundary. Meanwhile, since cleavage cracks propagate along specific crystallographic planes, HAGBs between cleavage planes enhance the ability to deflect cracks. These HAGBs are present at a significantly higher density within the lath structure. The blocky ferrite in GB lacks high-angle grain boundaries that can effectively resist crack propagation, with resistance relying mainly on prior austenite grain boundaries, as illustrated in Figure 12c. The brittle M-A constituents and coarse blocky ferrite greatly increase the risk of crack formation and propagation in granular bainite. In line with the weakest link theory, the primary cracks preferentially nucleate in granular bainite and propagate following the path of least resistance rapidly, as shown in Figure 12b_1_. The detrimental effects of massive M-A constituents on toughness can be summarized as follows: (i) massive M-A constituents fractured under the shear stress, leading to crack initiation; (ii) M-A constituents induce stress concentration, reducing the critical stress for cleavage crack propagation; (iii) microcracks, initially impeded by prior austenite grain boundaries, consume the resistance to the crack propagation, thereby facilitating the propagation of the primary crack; (iv) the unstable propagation of the fracture occurs through the coalescence of pre-formed micro-cracks within the GB matrix.

In contrast, the fracture behavior of the ISO-400 steel predominantly exhibits a plastic fracture mode, characterized by significant plastic deformation near the fracture surface and the great accumulation of geometrically necessary dislocations within the grains. Microcracks are effectively impeded by packet boundaries within the lath structure, as shown in Figure 12a_1_. The primary crack propagation path is narrow and frequently deflected by packet boundaries, block boundaries, and prior austenite grain boundaries, indicating that the fracture propagation energy is progressively dissipated by the high-angle grain boundaries. Small-sized M-A constituents have little influence on the toughness. The reduced interface between small-sized M-A constituents and ferrite matrix-relevant stress concentration. While the increase in the density of the M-A constituents of the ISO-400 steel may raise the stress concentration factor, the surrounding bainitic ferrite, characterized by its lath morphology, exhibits significantly higher shear resistance. The high-angle packet and block boundaries within the lath structure serve as effective barriers to crack propagation, thereby mitigating the negative effects of small-sized M-A constituents. In the quenching process, a reduction in the instantaneous cooling rate is inevitable. Since bainitic transformed at a high temperature will reduce low-temperature toughness, it is crucial to control the cooling rate around 450 °C, particularly in the central region of high-strength heavy plates. Slower cooling rates around 400 °C help promote the formation of lath bainite and improve the low-temperature toughness. This also explains the excellent properties observed at the 1/4 thickness position of heavy plates.

## 4. Conclusions

This study focuses on the isothermal bainite transformation of Ni-Cr-Mo steel, analyzing the bainite transformation content, morphology, structure, and crystallographic characteristics under different isothermal conditions, as well as their effects on subsequent martensitic transformation. Additionally, the study compares the low-temperature toughness of specimens subjected to different isothermal transformations and continuous cooling, examining the influence of crystallographic features and M-A constituents on low-temperature toughness. The main conclusions are as follows:

(1) As the transformation temperature decreases from 450 °C to 400 °C, the bainite transformation content increases from 20% to 100%, and the transformation rate markedly increases, exhibiting explosive transformation characteristics during the initial stage. Based on the K-M model, martensitic transformation curves were fitted for the CC-2 steel and ISO-400 steel. The results indicate that pre-transformed isothermal bainite induces the deformation of un-transformed austenite, promotes martensitic transformation, increases the martensitic transformation temperature, and enhances the transformation rate.

(2) The morphology of bainitic ferrite formed during isothermal transformation is strongly influenced by the transformation temperature. At 450 °C, the bainitic ferrite exhibits a blocky morphology, while at 400 °C, it appears predominantly lath-shaped. Carbon-enriched austenite, retained after isothermal holding, subsequently transforms into island-like M-A constituents below the martensite start temperature. As the transformation temperature increases from 400 °C to 450 °C, the average maximum length (L_max_) of these M-A constituents increases from 0.98 μm to 1.20 μm.

(3) For the ISO-450 steel, massive M-A constituents tend to crack during loading, promoting crack initiation and causing destructive brittle fracture. Additionally, the surrounding blocky ferrite fails to inhibit crack propagation, significantly reducing low-temperature toughness. Conversely, the fine M-A constituents in the ISO-400 steel exert minimal influence on the toughness and result in a more stable microstructure owing to their small size and the surrounding fine lath microstructure. In the quenching process of high-strength heavy plates, a reduction in cooling rate around 450 °C should be avoided.

## Figures and Tables

**Table 1:** The hardness and Charpy impact energy of the steels.

**Specimens**	**−60 °C AKV** **(J)**	**−** **80 °C AKV (J)**	**Hardness (HV** **10** **)**
CC-10	179	78	376
CC-2	191	180	367
ISO-400	193	181	333
ISO-450	94	59	338

**Table 2:** The numerical data of M-A constituents in the steels.

Specimens	Density of M-A (μm^−2^) ^+^	Average L_max_ (μm)	Average L_min_ (μm)	Average L_max_/L_min_
ISO-400	0.41	0.98	0.40	2.52
ISO-450	0.27	1.20	0.44	2.51

^+^ The statistical analysis of M-A constituent number and density in the ISO-450 sample was conducted based on those present within the GB regions.

## Data Availability

Data will be made available on request.

## References

[1] Sun X.J., Yuan S.F., Xie Z.J., Dong L.L., Shang C.J., Misra R.D.K. (2017). Microstructure-property relationship in a high strength-high toughness combination ultra-heavy gauge offshore plate steel: The significance of multiphase microstructure. Mater. Sci. Eng. A.

[2] Pan T., Wang X., Su H., Yang C. (2014). Effect of alloying element al on hardenabilitityand mechanical properties of micro-B treated ultra-heavy plate steels. Acta Metall. Sin..

[3] Fu T., Deng X., Tian X., Liu G., Wang Z. (2021). Experimental study on temperature drop during roller quenching process of large-section ultra-heavy steel plate. Sci. Prog..

[4] Liu W., Zhang S., Wang J. (2018). Experimental Study of Laminar Cooling Process on Temperature Field of the Heavy Plate. IOP Conf. Ser. Mater. Sci. Eng..

[5] Guo K., Pan T., Zhang N., Meng L., Luo X., Chai F. (2023). Effect of Microstructural Evolution on the Mechanical Properties of Ni-Cr-Mo Ultra-Heavy Steel Plate. Materials.

[6] Ranjan R., Singh S.B. (2021). Isothermal bainite transformation in low-alloy steels: Mechanism of transformation. Acta Mater..

[7] Hillert M. (1994). Diffusion in Growth of Bainite. Metall. Mater. Trans. A.

[8] Liu F., Zhang W., Lin X., Huang C., Liu F., Huang W., Wang P., Li X. (2020). Effect of isothermal temperature on bainite transformation, microstructure and mechanical properties of LSFed 300M steel. Mater. Today Commun..

[9] Li Q., Zhang Y., Li W., Huang X., Huang W. (2020). Improved Mechanical Properties of a Quenched and Partitioned Medium-Carbon Bainitic Steel by Control of Bainitic Isothermal Transformation. J. Mater. Eng. Perform..

[10] Huang Y., Li Q., Huang X., Huang W. (2016). Effect of bainitic isothermal transformation plus Q&P process on the microstructure and mechanical properties of 0.2C bainitic steel. Mater. Sci. Eng. A.

[11] Huang C., Zou M., Qi L., Ojo O.A., Wang Z. (2021). Effect of isothermal and pre-transformation temperatures on microstructure and properties of ultrafine bainitic steels. J. Mater. Res. Technol..

[12] Wang Z., Huang M.X. (2020). Optimising the strength-ductility-toughness combination in ultra-high strength quenching and partitioning steels by tailoring martensite matrix and retained austenite. Int. J. Plast..

[13] Guo K., Shi Z., Pan T., Luo X., Chai F. (2023). Structure and morphology of M-A constituent in the CGHAZ of a quenched and tempered ultra-low-carbon steel. J. Mater. Sci..

[14] Bhadeshia H.K.D.H. About calculating the characteristics of the martensite-austenite constituent. Proceedings of the International Seminar on Welding of High Strength Pipeline Steels.

[15] Takayama N., Miyamoto G., Furuhara T. (2018). Chemistry and three-dimensional morphology of martensite-austenite constituent in the bainite structure of low-carbon low-alloy steels. Acta Mater..

[16] Li Y., Baker T.N. (2010). Effect of morphology of martensite-austenite phase on fracture of weld heat affected zone in vanadium and niobium microalloyed steels. Mater. Sci. Technol..

[17] Li X., Fan Y., Ma X., Subramanian S.V., Shang C. (2015). Influence of Martensite-Austenite constituents formed at different intercritical temperatures on toughness. Mater. Des..

[18] Yang X., Di X., Liu X., Wang D., Li C. (2019). Effects of heat input on microstructure and fracture toughness of simulated coarse-grained heat affected zone for HSLA steels. Mater. Charact..

[19] Lan H.F., Du L.X., Misra R.D.K. (2014). Effect of microstructural constituents on strength-toughness combination in a low carbon bainitic steel. Mater. Sci. Eng. A.

[20] Huda N., Wang Y., Li L., Gerlich A.P. (2019). Effect of martensite-austenite (MA) distribution on mechanical properties of inter-critical Reheated Coarse Grain heat affected zone in X80 linepipe steel. Mater. Sci. Eng. A.

[21] Li X., Ma X., Subramanian S.V., Shang C., Misra R.D.K. (2014). Influence of prior austenite grain size on martensite-austenite constituent and toughness in the heat affected zone of 700MPa high strength linepipe steel. Mater. Sci. Eng. A.

[22] Ranjan R., Meena A. (2024). Influence of prior austenite grain size on martensite-austenite islands morphology and decomposition characteristics during two-step tempering in Mn-Ni-Mo steel. Mater. Today Commun..

[23] Jia S.J., Li B., Liu Q.Y., Ren Y., Zhang S., Gao H. (2020). Effects of continuous cooling rate on morphology of granular bainite in pipeline steels. J. Iron Steel Res. Int..

[24] Shipway P.H., Bhadeshiab H.K.D.H. (1995). The effect of small stresses on the kinetics of the bainite transformation. Mater. Sci. Eng. A.

[25] Richman R.H., Bolling G.F. (1971). Stress, Deformation, and Martensitic Transformation. Metall. Trans..

[26] Kabirmohammadi M., Avishan B., Yazdani S. (2016). Transformation kinetics and microstructural features in Low-Temperature Bainite after ausforming process. Mater. Chem. Phys..

[27] Li X., Shang C., Ma X., Subramanian S.V., Misra R.D.K., Sun J. (2018). Structure and crystallography of martensite–austenite constituent in the intercritically reheated coarse-grained heat affected zone of a high strength pipeline steel. Mater. Charact..

[28] Ramachandran D.C., Kim S.D., Moon J., Lee C.H., Chung J.H., Biro E., Park Y.D. (2020). Classification of martensite-austenite constituents according to its internal morphology in high-strength low alloy steel. Mater. Lett..

[29] Takayama N., Miyamoto G., Furuhara T. (2012). Effects of transformation temperature on variant pairing of bainitic ferrite in low carbon steel. Acta Mater..

[30] Qi X., Huan P., Wang X., Di H., Shen X., Sun Q., Liu Z., He J. (2021). Study on the mechanism of heat input on the grain boundary distribution and impact toughness in CGHAZ of X100 pipeline steel from the aspect of variant. Mater. Charact..

[31] Navarro-López A., Hidalgo J., Sietsma J., Santofimia M.J. (2017). Characterization of bainitic/martensitic structures formed in isothermal treatments below the Ms temperature. Mater. Charact..

[32] Yu Y., Zhao J., Wang X., Guo H., Xie Z., Shang C. (2025). Unraveling the significance of cobalt on transformation kinetics, crystallography and impact toughness in high-strength steels. Int. J. Miner. Metall. Mater..

[33] Chen J.H., Kikuta Y., Araki T., Yoneda M., Matsuda Y. (1984). Micro-fracture behaviour induced by m-a constituent (island martensite) in simulated welding heat affected zone of ht80 high strength low alloyed steel. Acta Metall..

[34] Lan L., Qiu C., Song H., Zhao D. (2014). Correlation of martensite-austenite constituent and cleavage crack initiation in welding heat affected zone of low carbon bainitic steel. Mater. Lett..

